# Seizure Freedom After Epilepsy Surgery and Higher Baseline Cognition May Be Associated With a Negatively Correlated Epilepsy Network in Temporal Lobe Epilepsy

**DOI:** 10.3389/fnins.2020.629667

**Published:** 2021-01-18

**Authors:** Elliot G. Neal, Mike R. Schoenberg, Stephanie Maciver, Yarema B. Bezchlibnyk, Fernando L. Vale

**Affiliations:** ^1^Department of Neurosurgery and Brain Repair, University of South Florida, Tampa, FL, United States; ^2^Department of Neurology, University of South Florida, Tampa, FL, United States; ^3^Department of Neurosurgery, Medical College of Georgia, Augusta University, Augusta, GA, United States

**Keywords:** epilepsy surgery, networks, resting fMRI, negative correlation, temporal lobe epilepsy

## Abstract

**Background:** Brain regions positively correlated with the epileptogenic zone in patients with temporal lobe epilepsy vary in spread across the brain and in the degree of correlation to the temporal lobes, thalamus, and limbic structures, and these parameters have been associated with pre-operative cognitive impairment and seizure freedom after epilepsy surgery, but negatively correlated regions have not been as well studied. We hypothesize that connectivity within a negatively correlated epilepsy network may predict which patients with temporal lobe epilepsy will respond best to surgery.

**Methods:** Scalp EEG and resting state functional MRI (rsfMRI) were collected from 19 patients with temporal lobe epilepsy and used to estimate the irritative zone. Using patients’ rsfMRI, the negatively correlated epilepsy network was mapped by determining all the brain voxels that were negatively correlated with the voxels in the epileptogenic zone and the spread and average connectivity within the network was determined.

**Results:** Pre-operatively, connectivity within the negatively correlated network was inversely related to the spread (diffuseness) of that network and positively associated with higher baseline verbal and logical memory. Pre-operative connectivity within the negatively correlated network was also significantly higher in patients who would go on to be seizure free.

**Conclusion:** Patients with higher connectivity within brain regions negatively correlated with the epilepsy network had higher baseline memory function, narrower network spread, and were more likely to be seizure free after surgery.

## Introduction

### Surgical Treatment of Temporal Lobe Epilepsy

Epilepsy is a common primary neurological disorder that affects 0.5–1% of the global population, of which 20–30% are refractory to medical management (Kwan and Brodie, 2000; Sander, 2003). For those patients who are refractory to medication, the next line of therapy involves some type of surgical intervention. If the epilepsy is focal and the epileptogenic zone can be localized to a temporal lobe using conventional techniques, which include electroencephalography (EEG), MRI, 18Fluoro-2-deoxyglucose positron emission tomography [(18F-FDG) PET], semiology, and neuropsychological testing, then a surgery can be planned to resect or ablate the hypothesized focus and disconnect the epileptogenic network. Surgeries that have been used to treat temporal lobe epilepsy include anterior temporal lobectomy, selective amygdalohippocampectomy, temporal lobectomy with amygdalectomy and minimal hippocampal resection, and stereotactic laser amygdalohippocampotomy (SLAH) (Schramm, 2008). Resective surgeries in the temporal lobe have been shown to result in seizure freedom in approximately two-thirds of patients, and an improved quality of life when compared to medical management alone (Wiebe et al., 2001; Bell et al., 2009; Engel et al., 2012; Vale et al., 2012). However, this still leaves the one-third of patients who undergo surgery in their temporal lobe who continue to have debilitating seizures.

### Network Analysis in Surgical Planning

When seizures recur, or are insufficiently controlled following surgery, it is commonly assumed that the surgical intervention was insufficient to resect, ablate, or disconnect the epileptogenic brain region(s). This problem may arise when the epileptogenic zone is knowingly spared due to concerns for post-operative neurocognitive or neurological function, or when the epileptogenic zone is incompletely evaluated by the pre-operative work-up. Several authors have proposed a network model for epilepsy, whereby the pre-operative work-up is directed toward elucidating the connectivity within and extent of the epileptogenic network which is both necessary and sufficient for post-operative seizure control, as well as the relationship of this network with networks underlying neurocognitive function (Bartolomei et al., 2008; Bernhardt et al., 2013; Gonzalez et al., 2019; Morgan et al., 2019). Typically, this consists of invasive monitoring using intracranial EEG depth electrodes (Chauvel et al., 2019). However, such studies are labor-intensive, take time, and carry an inherent risk, leading to efforts to devise non-invasive strategies for modeling epilepsy-related functional networks that can be used to predict who is more likely to be relieved of seizures.

### Non-invasive Epilepsy Network Modeling

We previously developed a network modeling algorithm that uses rsfMRI and surface EEG to generate a hypothesized epileptogenic zone non-invasively. This algorithm then generates a network of other regions with activation patterns that have a high degree of positive correlation with the epileptogenic zone in patients with temporal lobe epilepsy (Neal et al., 2018). With this model, we showed that the degree of spread of this positively correlated “epilepsy network” in patients with TLE was associated with relatively worse outcomes both in rates of seizure freedom and in measures of cognition including executive function and verbal memory (Neal et al., 2019). Furthermore, we showed that greater disconnection of this network after surgery was associated with a higher likelihood of seizure freedom (Neal et al., 2020). These results suggest that the positively correlated epilepsy network may be associated with impaired cognition in patients with temporal lobe epilepsy and that disconnection of this network may impede the generation and propagation of seizures. Thus far, the only network that we have studied is the positively correlated epilepsy network.

### Negative Correlation in Network Analysis

**FIGURE 1:**
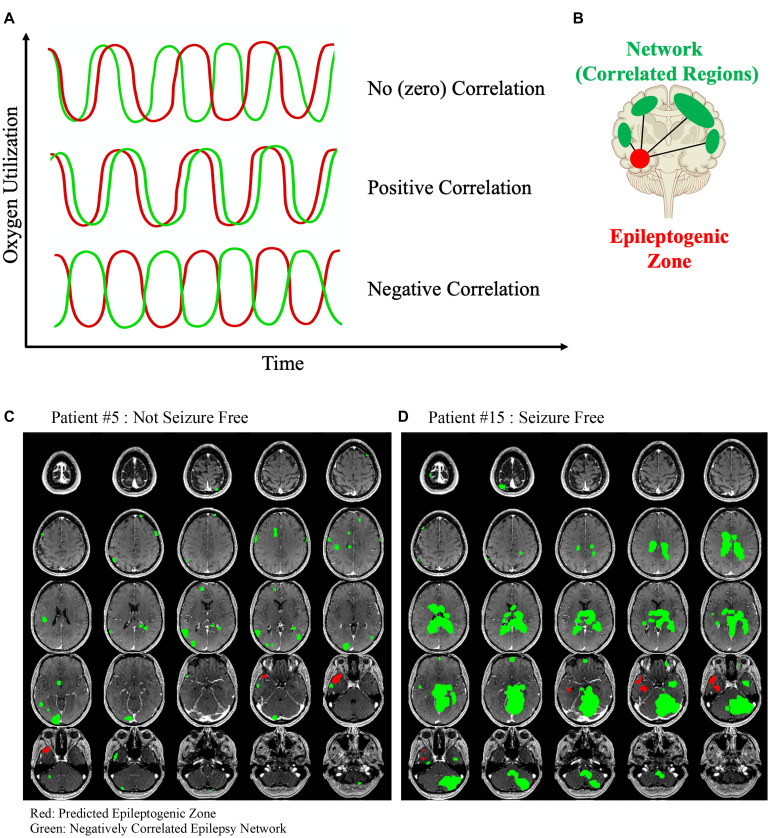
**(A)** Three different example time series data from two hypothetical rsfMRI voxels are shown. First, the oxygen utilization over time between two hypothetical voxels is unrelated and therefore the correlation between the two is approximately zero. Next, the time series of each voxel behaves similarly and therefore have a positive correlated coefficient indicating that they are functionally similar and likely intraconnected. Finally, two voxels that behave antagonistically but are in-phase would have a negative correlation coefficient and we hypothesized that this relationship may represent a different type of functional connectivity than the positive correlated voxels. **(B)** The negatively correlated epilepsy network is then generated between the voxels in the epileptogenic zone and all of the other brain voxels with a below-threshold correlation coefficient. Two example patients are shown. **(C)** Patient #5 was not seizure free after surgery, and **(D)** Patient #15 was seizure free. The negatively correlated network is shown in green, and the hypothesized epileptogenic zone, generated from surface EEG, is shown in red. The lack of a clear organized negatively correlated network is obvious in the first patient, and the only visible areas are likely a contribution of the noisy rsfMRI signal and reflects the clear difference that exists in this network between patients who are more likely to be seizure free after surgery compared to those who are not.

A positive correlation in activation patterns indicates that one or more brain regions are likely connected in some way. However, the opposite case may also be true: negatively correlated brain regions may also be functionally connected (Figure 1A). For example, if a particular neuron or group of neurons has an inhibitory effect on its target, then every time the first neuron fires then the target would fire less frequently. Therefore, a positive activation in one region would be directly associated with a deactivation in the connected area (Figure 1B). Mathematically, this can be determined as a negative correlation value (Pearson correlation coefficient <0). Negative correlation between nodes of the DMN (default mode network) have been observed with relation to task positive networks in healthy patients (Uddin et al., 2009). Negative correlation has, for the most part, been studied in relation to generalized epilepsies, with findings being mostly related to the behavior of the DMN. Antagonism between the dorsal attention network, salience network, and DMN was shown to be related to impaired executive and attention function in patients with absence seizures (Li et al., 2015). A similar relationship between the task positive network and the DMN in children with benign childhood epilepsy with centrotemporal spikes was found when compared to a control group (Luo et al., 2016). Patients with idiopathic generalized epilepsy have been found to have segregation and negative correlation between regions of the DMN (McGill et al., 2012).

In temporal lobe epilepsy, network studies utilizing negative correlation have shown decreased connectivity in the temporal lobes and a compensatory increase in the default mode network connectivity over time (Zhang et al., 2010). In a SPECT-based network study in temporal lobe epilepsy examining the positive and negative network correlations, the authors found that alterations in consciousness during seizures were associated with increased cerebral blood flow (CBF) in the temporal lobes and midline subcortical structures, which was negatively correlated with CBF in the frontal and parietal association cortices (Blumenfeld et al., 2004). In a small study of patients who underwent surgery for temporal lobe epilepsy, a similar result was found. rsfMRIs performed in these patients demonstrated a negative correlation between the temporal lobe and widespread cortical and subcortical regions compared to controls (Morgan et al., 2010). These regions included the thalamus, brainstem, frontal regions, and parietal regions, whereas the control group did not have this same widespread network of negatively correlated regions.

### Objective

In the current study we hypothesize that the network consisting of brain regions negatively correlated with the epileptogenic zone can be determined in patients with temporal lobe epilepsy undergoing surgery and intraconnectivity and spread of that network may be predictive of seizure freedom and neuropsychological outcomes after surgery.

## Materials and Methods

### Patient Demographics

All reported data followed the Strengthening the Reporting of Observational studies in Epidemiology (STROBE) guidelines for observational trials and the protocol and informed consent was approved by our university’s Institutional Review Board (IRB). Epileptic networks were modeled in nineteen patients with unilateral TLE who underwent open microsurgery directed toward the anterior/mesial temporal lobe. The patients included in this study represent a series of patients with TLE who signed consent and agreed to participate in this study between May 2017 and March 2020. They underwent this pre-operative network assessment and surgery at our tertiary referral center (Table 1). Exclusion criteria included all patients who had less than 1 year of follow up, pre-pubertal children (youngest patient was 17 years old at the time of surgery), and patients with any brain lesion except for mesial temporal sclerosis. Each patient underwent a standard pre-surgical evaluation for epilepsy surgery including MRI, epilepsy monitoring unit (EMU) video-EEG, Wada testing, (18F-FDG) PET, and neuropsychological testing. Surgery planning and post-surgical evaluation not related to network analysis were conducted by a care team blinded to the epileptic network modeling results.

**TABLE 1:** Demographics.

Patient number	Gender	Age at surgery	Anti-seizure medications (at surgery)	Surgery side	Dominant hemisphere (Wada)	Seizure free
1	Male	26	Levetiracetam, Lamotrigine	Left	Left	No
2	Male	17	Lamotrigine	Left	Right	No
3	Female	26	Vimpat, Aptiom	Right	Left	No
4	Female	35	Carbamazepine, Lamotrigine	Left	Left	No
5	Female	32	Levetiracetam, Topiramate	Left	Right	No
6	Female	40	Zonisamide	Right	Left	No
7	Female	36	Lamotrigine	Left	Left	No
8	Female	47	Gabapentin	Left	Left	Yes
9	Male	23	Levetiracetam	Left	Left	Yes
10	Female	34	Lacosamide, Levetiracetam	Left	Right	Yes
11	Female	19	Lamotrigine, Levetiracetam, Topiramate	Right	Left	Yes
12	Female	40	Oxcarbazepine	Right	Left	Yes
13	Male	26	Lacosamide, Levetiracetam, Topiramate	Left	Left	Yes
14	Female	33	Brivaracetam, Lacosamide, Lamotrigine	Left	Left	Yes
15	Male	32	Lacosamide	Left	Left	Yes
16	Female	28	Lamotrigine, Levetiracetam, Perampanel, Zonisamide	Left	Left	Yes
17	Female	24	Levetiracetam	Left	N/A	Yes
18	Male	25	Perampanel, Oxcarbazepine	Left	Left	Yes
19	Female	53	Lamotrigine	Left	Left	Yes

### Data Acquisition

Electroencephalography and rsfMRI were obtained on separate visits non-concurrently as part of the pre-surgical evaluation. No patient had a clinical seizure during the duration of the rsfMRI acquisition, though we cannot say with absolute certainty that patients did not have subclinical seizures during the acquisition period. In our cohort there were no patients that had interictal discharges more frequent than one per hour according to subsequent epilepsy monitoring unit results. EEG was acquired with 24 scalp electrodes in a standard International 10–20 configuration during the pre-operative EMU (Epilepsy Monitoring Unit) session. rsfMRI was conducted in a 3-Tesla MRI with a blood oxygenation level dependent (BOLD) MRI sequence. rsfMRI was acquired with the patient lying supine with eyes closed. The rsfMRI sequence consisted of a single 5-min acquisition with parameters as follows: echo time (TE) of 35 ms, repetition time (TR) of 3000 ms, and a voxel size of 4 mm × 3.75 mm × 3.75 mm. Volumetric T1-weighted thin slice (1.5 mm) MRI [Repetition Time (TR) 6.484, Echo Time (TE) 2.958, Inversion Time 18, Flip Angle 15, Field of View (FOV) 240 mm × 240 mm] was acquired during the same session. The post-operative MRI was conducted 4 months after the surgery to allow the acute surgery-related MRI signal to dissipate and not affect the results.

### Network Modeling

The epilepsy network for each patient was modeled similar to what is previously described (Neal et al., 2018). Briefly, all MR image sets were motion corrected, smoothed, and transformed into Montreal Neurological Institute (MNI) space using the six-parameter rigid body spatial transformation algorithm and co-registered using SPM12 (Wellcome Department of Imaging Neuroscience, University College London, United Kingdom). The motion correction step was performed by least-squares approach to realign all images in the rsfMRI set to the first image in the series to reduce the effect of patient motion on the post-processing analysis. The Gaussian smoothing kernel full width at half maximum was 8 mm in all directions. The scalp EEG data were filtered to remove non-physiologic frequencies and cropped to include only the inter-ictal or ictal signals identified by a blinded neurophysiologist (MATLAB 2019b, Natick, MA, United States). A band pass filter was applied between 1 and 100 Hz, and a notch filter was applied at 60 Hz. Ictal and inter-ictal source discharges were localized by first generating a transformed mesh from the thin-slice T1-weighted MRI sequence. Then, cortical dipoles were modeled using a forward computation that was followed by an empirical Bayesian approach to inverse reconstruction, localizing the theoretical evoked response (SPM12). This process was used to generate a hypothesized epileptogenic or irritative zone source volume from both interictal and ictal discharges, which was co-registered to the rsfMRI in MNI space. The combined ictal and interictal regions in the temporal lobe ipsilateral to the planned surgical resection were included in the next steps of analysis for generating a model of the network.

The rsfMRI time-series signature was extracted from the epileptogenic zone volume and scaled to the global signal average to adjust for differences between scans. Intra-axial image voxels were extracted using a brain mask and compared to the voxels in the hypothesized epileptogenic zone to generate a Pearson correlation coefficient for each voxel with respect to the epileptogenic zone. In previous studies using this algorithm, the epilepsy network was defined as the collection of voxels that had an above-threshold Pearson correlation, with the threshold defined as the average Pearson correlation coefficient for each patient, so that all volumes generated were positively correlated with the epileptogenic zone. This type of analysis was done in the present study to generate an epilepsy network, but the primary aim of this study was to investigate the negatively correlated regions, so the inverse epilepsy network was defined as the voxels that had a Pearson correlation value less than −0.4. The threshold −0.4 was selected to standardize the analysis between patients, which has been shown to have a high sensitivity for detecting nodes within brain networks (van den Heuvel et al., 2008; Shen et al., 2013). Similar to our prior studies, intraconnectivity within this unique network was calculated by determining the correlation matrix for all the voxels involved in the inverse epilepsy network and computing an average value. This intraconnectivity is calculated from the within-network functional connectivity between voxels inside the negatively correlated epilepsy network, averaged across the entire network. To clarify, the intraconnectivity is not calculated between the negatively correlated network voxels and the hypothesized epileptogenic zone. Similarly, post-operative connectivity within the modeled network was determined by calculating the mean Pearson connectivity coefficient within the network when the same set of voxels were overlaid on the post-operative rsfMRI image set. The spread of the negatively correlated epilepsy network was defined as the median Euclidean distance of each voxel within the network from the centroid of the hypothesized epileptogenic zone generated from the EEG source localization. The network was mapped without prior knowledge of any parameters in the rsfMRI for each patient and is not related to any anatomical or functional atlas. Two examples, one of a negatively correlated that is more diffuse (Figure 1C) and one that is highly organized and synchronized (Figure 1D) are shown. All network maps and hypothesized epileptogenic zones are also included in Supplementary Figure 1.

As a control comparison, a random ROI was used to generate a random negatively correlated network using the exact same method as described above. The random ROI used was in the occipital lobe and had a size of 907 voxels, similar to the average seed size for the hypothesized epileptogenic zone seeds. The same metrics of network spread and intraconnectivity were calculated as described above.

### Neuropsychological Assessment

Pre-operatively, 19 patients had comprehensive neuropsychological assessment following NIH Epilepsy common data elements recommendations that quantify aspects of cognition including declarative memory, attention/executive, language, and visuoconstructional functions as well as general intellectual ability. Quality of life and mood status was also obtained. Pre- and post-operative data were available for a subset of patients (*n* = 13) because data from the remaining patients are still being collected and processed. Testing and scoring were conducted by clinicians blinded to the network modeling results. Subtests of the Wechsler Memory Scale-4th Ed. (WMS-IV) analyzed immediate or delayed logical memory (LM-I and LM-II) and immediate or delayed visual reproduction (VR-I and VR-II), a measure of visual memory. The Rey Auditory Verbal Learning Test short-delay (RAVLT Trials 6) and long-delay (RAVLT 7) was used to measure auditory-verbal memory, rate of learning, learning strategies, retroactive and proactive interference, the presence of confabulation in memory processes, retention of information, and differences between learning and retrieval. Both RAVLT 6 and 7 and LM-I and II are tests that measure verbal memory. Verbal fluency was measured using the Controlled Oral Word Association (FAS) and semantic fluency was measured using the Animal Naming Test. Word retrieval was measured using the Boston Naming Test (BNT). The Ruff Figural Fluency Test (RFFT) evaluated non-verbal mental flexibility, initiation, planning, and divergent reasoning. Finally, each patient completed the Wechsler Adult Intelligence Scale – 4th Ed (WAIS-IV) prorated full-scale intelligence index. Raw scores for all neuropsychological tests except for WAIS-IV IQ scores were used in analyses. We obtained a “difference score” – defined as the post-operative score minus the pre-operative score – such that higher difference scores correlate to relatively higher function post-operatively, and conversely lower scores representing a drop in these objective measures of cognition.

### Statistical Analysis

A two-sample *t*-test was used to compare independent groups with continuous variables. *P*-values less than α = 0.05 were considered significant. Spearman Rho correlation analysis was used to compare network connectivity to neuropsychometric testing results with a Bonferroni correction for multiple comparisons. Network modeling statistical tests were conducted using IBM SPSS Statistics Version 26 (IBM Corp., Armonk, New York, United States).

### Data Availability

The data that support the findings of this study are available from the corresponding author, upon reasonable request.

## Results

### Demographics

In the cohort of 19 patients with unilateral TLE, five (26%) were male and 14 (74%) were female. All underwent the complete phase 1 evaluation described in the methods, with three (16%) of those patients undergoing subsequent phase II invasive monitoring (stereo-electroencephalography or subdural strips/grids) for further clarification of epileptogenic focus localization. Fifteen (79%) patients were determined to have seizures originating from the left temporal lobe with the remaining four (21%) having seizures in the right temporal lobe. All patients underwent microsurgical resection with either selective amygdalohippocampectomy (*n* = 13) or temporal lobectomy with amygdalectomy and minimal hippocampal resection (*n* = 6). Pre-operatively, ten (53%) patients had suspicion of mesial temporal sclerosis on MRI, and none of the patients had any other MRI lesions elsewhere in the brain. After surgery, the same ten (53%) had tissue specimen proven hippocampus sclerosis. All patients were followed for at least 1 year after surgery, and with an average time to follow-up of 24 months. Demographic information is also shown in Table 1. All 19 patients had the negatively correlated epilepsy network mapped, and the surgery planning team was blinded to the results of the network result. In addition, the seed size was determined for each patient and found to be not significantly different between the seizure free and the not seizure free group (810 vs. 858 voxels, *p* = 0.7461). The seed size for each patient is included in Supplementary Figure 1.

### Highly Intraconnected Negatively Correlated Epilepsy Networks Are Less Widespread

The relationship between the positively correlated network and outcomes after surgery has been assessed in previous papers, and this analysis was performed to determine if these two networks are independent from each other to prove that the negatively correlated epilepsy network is a unique prognostic tool. Across all patients, the degree of connectivity within each of these networks was not significantly correlated (*R* = −0.255, *p* = 0.293), suggesting that connectivity within the negatively correlated epilepsy networks and within the positively correlated epilepsy networks are independent variables. Next, the connectivity between the voxels of the negatively correlated network (the “intraconnectivity” of the network) was compared to the average connectivity between the individual voxels to the epileptogenic zone (negative correlation to the seed volume) and found to be not significantly related (*R* = 0.130, *p* = 0.841). This suggested that the intraconnectivity metric is independent from the negative correlation observed between the voxels of the network and the epileptogenic zone.

**FIGURE 2:**
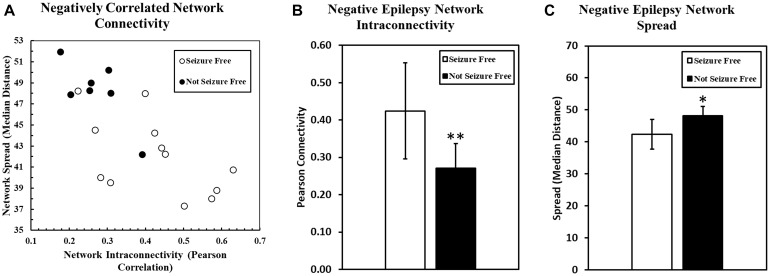
**(A)** The relationship between the spread of the negatively correlated epilepsy network and the intraconnectivity of that network is shown. Intraconnectivity of the network was calculated by determining the average correlation value between each network voxel and all of the other voxels in the network. These data suggest that the intraconnectivity of the negatively correlated epilepsy network is inversely related to the spread of that network. Patients who are seizure free after surgery are more likely to have a highly connected and less spread out network. Two bar charts are shown here, with one **(B)** comparing the connectivity of the negatively correlated epilepsy network between patients who were seizure free after surgery and those who were not. The same groups are compared in the second bar chart **(C)** comparing the geographical spread of the network, with higher numbers representing a more diffuse, spread out network. (^∗^*p* < 0.05, ^∗∗^*p* < 0.01).

The intraconnectivity of the negatively correlated epilepsy network was significantly correlated to the spread of the network, such that the more highly intraconnected the network, the less it was spread out across the brain (*R* = −0.6905, *p* = 0.0011). A scatter plot of the network spread and intraconnectivity is shown in Figure 2A.

### Correlation With Improved Baseline Memory Function and Decline in Post-operative Naming Function

We found that both immediate and delayed visual memory function were higher in patients with a more intraconnected negatively correlated network (VRI *R* = 0.561, *p* = 0.012; VRII *R* = 0.549, *p* = 0.015). No other significant correlations were found pre-operatively.

Neuropsychological function was also measured post-operatively for comparison to the pre-operative level. We observed a negative correlation between the difference score for naming and the pre-operative connectivity within the negatively correlated epilepsy network, suggesting that patients with more intraconnected negatively correlated epilepsy networks are more likely to experience a larger decrease in their naming function (BNT *R* = −0.590, *p* = 0.045). It should be noted that the pre-operative performance on the same test was not significantly correlated with negatively correlated epilepsy network connectivity (*R* = 0.356, *p* = 0.134), suggesting that the correlation of the difference in score was not likely related to a baseline deficit in the patients with less intraconnected networks.

### Negatively Correlated Epilepsy Network Connectivity and Seizure Freedom

Seizure outcome was monitored at all stages of follow up after surgery, but the Engel classification used in the present study was determined at the most recent follow up for each patient. As of the most recent follow-up (range: 14–36 months), seven patients (37%) were not seizure free (Engel Class II, III, IV), and 12 patients (63%) were seizure free (Engel Class I). The connectivity within the pre-operative positively correlated epilepsy network was not significantly different between patients that were seizure free and those that were not (*p* = 1.00). However, the pre-operative connectivity within the negatively correlated network was significantly higher in patients who went on to be seizure free (*p* = 0.0095) (Figure 2B). Also, spread of the network (median distance) was significantly lower in patients who were seizure free compared to those who were not (*p* = 0.0083) (Figure 2C).

The control network maps consisting of the negatively correlated networks generated from a random ROI were analyzed in the same way and the results are shown in Supplementary Figure 2. In this control network comparison, the spread of the network was not correlated with within-network intraconnectivity (*R* = 0.2338, *p* = 0.3354). The spread of the randomly generated negative network was not significantly higher in the patients who were not seizure free (*p* = 0.1570), and the intraconnectivity was also not significantly different between the two groups (*p* = 0.2901) (Supplementary Figure 2).

**FIGURE 3:**
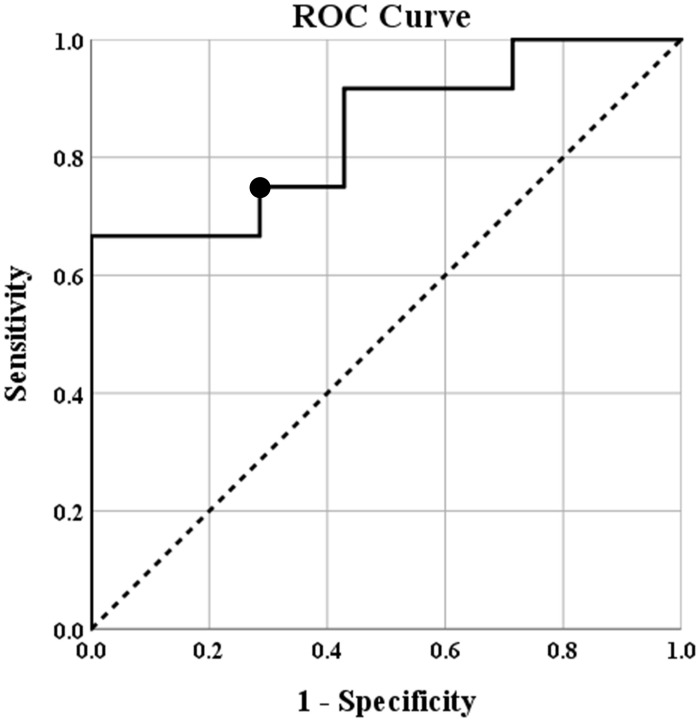
Receiver operating characteristics (ROC) curve showing the predicted specificity and sensitivity of various thresholds of intraconnectivity of the negatively correlated epilepsy network. The diagonal line represents y = x reference line, the second line shows the sensitivity and specificity of predicting seizure freedom at incremental thresholds, and the circle marker represents the threshold (0.2728) that maximized sensitivity (75%) and specificity (71%) of this threshold to predict seizure freedom after surgery. The area under the curve was determined to be 0.845 with the 95% confidence interval ranging from 0.670 to 1.00.

The predictive value of the negatively correlated epilepsy network was also investigated. A receiver operating characteristics (ROC) curve was created. The area under the curve was determined to be 0.845 with the 95% confidence interval ranging from 0.670 to 1.00. The connectivity threshold that maximized specificity and sensitivity was (0.2728) (sensitivity 75%, specificity 71%). A threshold of 0.2136 maximized sensitivity (specificity 57%, sensitivity 92%) and a threshold of 0.3955 maximized specificity (specificity 100%, sensitivity 67%). The ROC curve is shown in Figure 3.

## Discussion

### Is Negatively Correlated Epilepsy Network Connectivity a Good Sign?

In this paper, we defined and analyzed the “negatively correlated epilepsy network” – a novel network that is negatively correlated with the epileptogenic zone in 19 patients with temporal lobe epilepsy who underwent open microsurgery. Pre-operatively, we found that patients with highly intraconnected negatively correlated epilepsy networks were more likely to have higher baseline verbal and logical memory. Furthermore, the same network was more highly intraconnected in patients that would go on to be seizure free after epilepsy surgery. High intraconnectivity and less spread in the negative network was interpreted as a network that is more organized, because it is more focal in its location in the brain, and all brain regions involved in the network work together in synchrony. Put simply, a more organized and homogeneous negatively network appears to be a good prognostic factor in patients with temporal lobe epilepsy undergoing surgery. Importantly, when these results were compared to the same relationship in networks generated from a random ROI, it was shown that there was no significant relationship between intraconnectivity, spread, and seizure freedom after surgery. This indicates that the negatively correlated epilepsy network generated from a seed volume consisting of the hypothesized epileptogenic zone is unique and appears to be uniquely correlated with a clinically significant outcome after surgery. It was shown that this simple, non-invasive test may be useful as a prognostic test to predict seizure freedom.

The network consists of a group of brain regions that are unique in that they have a negatively correlated rsfMRI signature compared to the time series of the epileptogenic zone as determined based on the irritative zone identified on the pre-operative scalp EEG. We have previously described a positively correlated “epilepsy network,” defined as the network of regions with activation patterns that have a high degree of positive correlation with the epileptogenic zone in patients with temporal lobe epilepsy, and we hypothesized that the negatively correlated regions might be similarly important in understanding the brain function of the patient with temporal lobe epilepsy. In our previous study, whereas the spread of the positively correlated network was associated with worse baseline function, baseline connectivity within the network was not predictive of which patients would benefit from surgery. The degree of disconnection in that network after surgery was, however, associated with more seizure free outcomes. In contrast, the data presented in the current study suggest that baseline connectivity within the negatively correlated epilepsy network help to select for patients who are most likely benefit from temporal lobe surgery.

A negative correlation, represented by a negative Pearson correlation value, has been associated with brain regions that act antagonistically (Uddin et al., 2009). Therefore, it is possible that the negatively correlated epilepsy network that is more intraconnected may antagonize the function of the epileptogenic zone in a way that benefits the patient both pre- and post-operatively. Perhaps the negatively correlated network acts opposed to the epileptogenic zone during inter-ictal periods and controls the negative effect that the aberrant activity in the epileptogenic zone have on normal cognition. This may explain why our cohort of patients with TLE had improved performance on neuropsychological evaluations (specifically in memory and executive function) that scaled directly with the degree of connectivity within the negatively correlated network.

Studies involving simultaneous acquisition of EEG and fMRI data have been conducted previously by several groups and involve an analysis of negatively contrasted images that is somewhat similar to the present study but with important differences. These studies demonstrated that such an analysis can be used to correlate hemodynamic changes in the brain with the epileptogenic zone, and have shown that resection of the epileptogenic zone, when closely associated with brain regions exhibiting changes in BOLD activation, is positively correlated with post-operative seizure control (Thornton et al., 2010; van Houdt et al., 2013; Coan et al., 2016). While similar to our approach, it is a crucial difference to note that in these studies EEG and rsfMRI data were acquired simultaneously, such that connectivity measurements were analyzed at the same time that epileptic discharges were detected. As such, these studies generate an ictal pattern of functional connectivity which can then be correlated to the electrophysiological data in a time-locked fashion. However, when data is acquired in this fashion, the discharges likely cause a momentary change in the connectivity patterns which is helpful for detecting the locally connected epileptic network. In the current study, we elected instead to measure EEG and rsfMRI non-concurrently. As such, our connectivity data reflects functional connectivity patterns existing in the interictal state, might well be different than those observed during a seizure or epileptic discharge, and could reflect network connectivity that is related to background normal function of the brain that is known to be impaired in patients with temporal lobe epilepsy. Alternatively, the results could represent a different set of network relationships influential in background memory and executive processing.

### Is There Is Any Value in Epilepsy Surgery Planning?

Since higher connectivity within the negatively correlated epilepsy network was associated with a higher likelihood of seizure freedom post-operatively, the prognostic value of this single metric was also investigated. Within our cohort, an average connectivity within the negatively correlated epilepsy network of 0.2728 predicts seizure freedom with a specificity of 71% and a sensitivity of 79%. Clinically, this tool is non-invasive and easy to use. The negatively correlated network is created automatically with standard EEG and MRI technology that is available during a normal phase I epilepsy surgery evaluation, and so can be readily integrated into the standard pre-operative workflow of any comprehensive epilepsy center.

### Future Studies

This is still preliminary data and any hypotheses about the exact mechanism of this negatively correlated epilepsy network still need to be confirmed in future studies by applying the thresholds calculated with the ROC analysis in an out-of-sample test group to see if the prediction holds. Also, there has been much work on multivariate predictive models in epilepsy surgery (Spencer et al., 2005; Jehi et al., 2015). In the future this unique connectivity metric may be able to add to the predictive value of these models. We will continue to collect data and build a larger database in the hopes of finding more subtle changes between our subgroups. We also plan to start including additional data such as semiology and PET scan to better define network characteristics. Magnetoencephalography and SEEG combined studies have also shown that a multimodal approach using those methods can help to further refine the localization of the epileptogenic zone, and we will explore the possibility of adding this type of data into future studies (Badier et al., 2017; Pizzo et al., 2019). These network imaging results will be discussed in the multi-disciplinary evaluation for epilepsy surgery in an effort to generate prospective, out-of-sample data to substantiate the conclusions of this study. The goal of this type of research would be to strengthen the argument that network analysis can be a valuable tool in predicting which patients will benefit from resective surgeries.

### Limitations

While not detracting from the results presented here, some limitations to the applicability of this data should be considered. First, this is a prospective but small cohort and therefore represents preliminary data. Future studies will aim to address this limitation, as described above. Furthermore, the rsfMRI image sets collected in this study are from a single session and may therefore have lower signal-to-noise ratios than comparable data taken across multiple sessions or for longer acquisition times. It is well known that surface EEG is limited in its precision and accuracy in estimating an epileptogenic zone, and the aim of this study was to determine if the utility of scalp EEG in defining an EZ and predicting post-operative surgical outcomes can be enhanced with the addition of different measures of functional connectivity. The attempt is not to localize the EZ with 100% accuracy, but to determine if clinically useful properties can be extracted from functional connectivity data acquired non-invasively. Finally, when undergoing rsfMRI imaging, we did not control for the presence of anti-epileptic drugs. It is possible that these drugs may inhibit certain neuronal processes and affect rsfMRI signal patterns, and it is unknown how these drugs would affect our results.

## Conclusion

In this study, preliminary data was shown, and patterns of connectivity were explored within a newly defined “negatively correlated epilepsy network” in patients with refractory temporal lobe epilepsy undergoing surgery. In the network, defined as those areas of the brain that had a below-threshold negative Pearson correlation with the hypothesized epileptogenic zone estimated from surface EEG, intraconnectivity was directly related to pre-operative performance on objective neuropsychological evaluation of verbal and logical memory. Also, pre-operative negatively correlated epilepsy network connectivity was directly related to the likelihood of being seizure-free after surgery. These findings together suggest a benefit to the patient both pre- and post-operatively if they had a highly connected negatively correlated epilepsy network, which can be determined using commonly available, non-invasive methods (EEG and rsfMRI). Even though this study is limited in size, the preliminary, proof of concept findings suggest a novel methodology of detecting negatively correlated brain regions may be useful in deciding which patients would most likely benefit from surgery. These data represent a step toward proving the efficacy of non-invasive network mapping and should stimulate future exploration into the utility and value of such an algorithm in predicting outcomes after surgery.

## Data Availability Statement

The raw data supporting the conclusions of this article will be made available by the authors, without undue reservation.

## Ethics Statement

The studies involving human participants were reviewed and approved by the University of South Florida Institutional Review Board. The patients/participants provided their written informed consent to participate in this study.

## Author Contributions

All authors listed have made a substantial, direct and intellectual contribution to the work, and approved it for publication.

## Conflict of Interest

EN and FV are inventors and hold a US patent (#10,588,561) for the network modeling algorithm used in this study. The remaining authors declare that the research was conducted in the absence of any commercial or financial relationships that could be construed as a potential conflict of interest.

## Supplementary Material

Supplementary Figure 1 All hypothesized epileptogenic seed volumes are shown in red for each patient and the seed volume size is also given. The green volumes represent the negatively correlated epilepsy network for each patient. **(A)** Hypothesized epileptogenic zones and negatively correlated epilepsy networks for all patients who were not seizure free. **(B)** Seed volumes and negatively correlated epilepsy network are shown for all patients that were seizure free.

Supplementary Figure 2 A randomly generated ROI was used to create a negatively correlated network using the same algorithm as described in the “Materials and Methods” section. **(A)** No significant relationship between network spread and intraconnectivity is seen when the negatively correlated epilepsy network maps are analyzed. **(B)** No significant difference in network intraconnectivity or **(C)** spread was found between patients that were seizure free after surgery and those who were not.

## Abbreviations

(18F-FDG) PET: 18Fluoro-2-deoxyglucose positron emission tomography
BOLD: Blood oxygenation level dependent
BNT: Boston Naming Test
COWAT-FAS: Controlled Oral Word Association Test
ECoG: Electrocorticography
EEG: Electroencephalography
EMU: Epilepsy Monitoring Unit
MNI: Montreal Neurological Institute
rsfMRI: Resting state functional MRI
RAVLT6: Rey Auditory Verbal Learning Test, Trial 6
RAVLT7: Rey Auditory Verbal Learning Test, Trial 7
RFFT: Ruff Figural Fluency Test-unique designs
TLE: Temporal lobe epilepsy
FSIQ: Full Scale Intelligence Quotient: Wechsler Adult Intelligence Scale-4th Ed.
LM-I: Logical Memory Immediate recall subtest: Wechsler Memory Scale-4th Ed.
LM-II: Logical Memory Delayed recall subtest: Wechsler Memory Scale-4th Ed.
VR-I: Visual Reproduction Immediate Recall subtest: Wechsler Memory Scale-4th Ed.
VR-II: Visual Reproduction Delayed Recall subtest: Wechsler Memory Scale-4th Ed.
