# Supplementary material for: Seizure Freedom After Epilepsy Surgery and Higher Baseline Cognition May Be Associated With a Negatively Correlated Epilepsy Network in Temporal Lobe Epilepsy
Source: Front Neurosci. 2021 Jan 18;14:629667. doi: 10.3389/fnins.2020.629667 (PMC7874020; doi:10.3389/fnins.2020.629667)
Supplement: Supplementary Figure 1 — All hypothesized epileptogenic seed volumes are shown in red for each patient and the seed volume size is also given. The green volumes represent the negatively correlated epilepsy network for each patient. (A) Hypothesized epileptogenic zones and negatively correlated epilepsy networks for all patients who were not seizure free. (B) Seed volumes and negatively correlated epilepsy network are shown for all patients that were seizure free. [file Data_Sheet_1.docx]

**Supplementary Figure 1:**

**
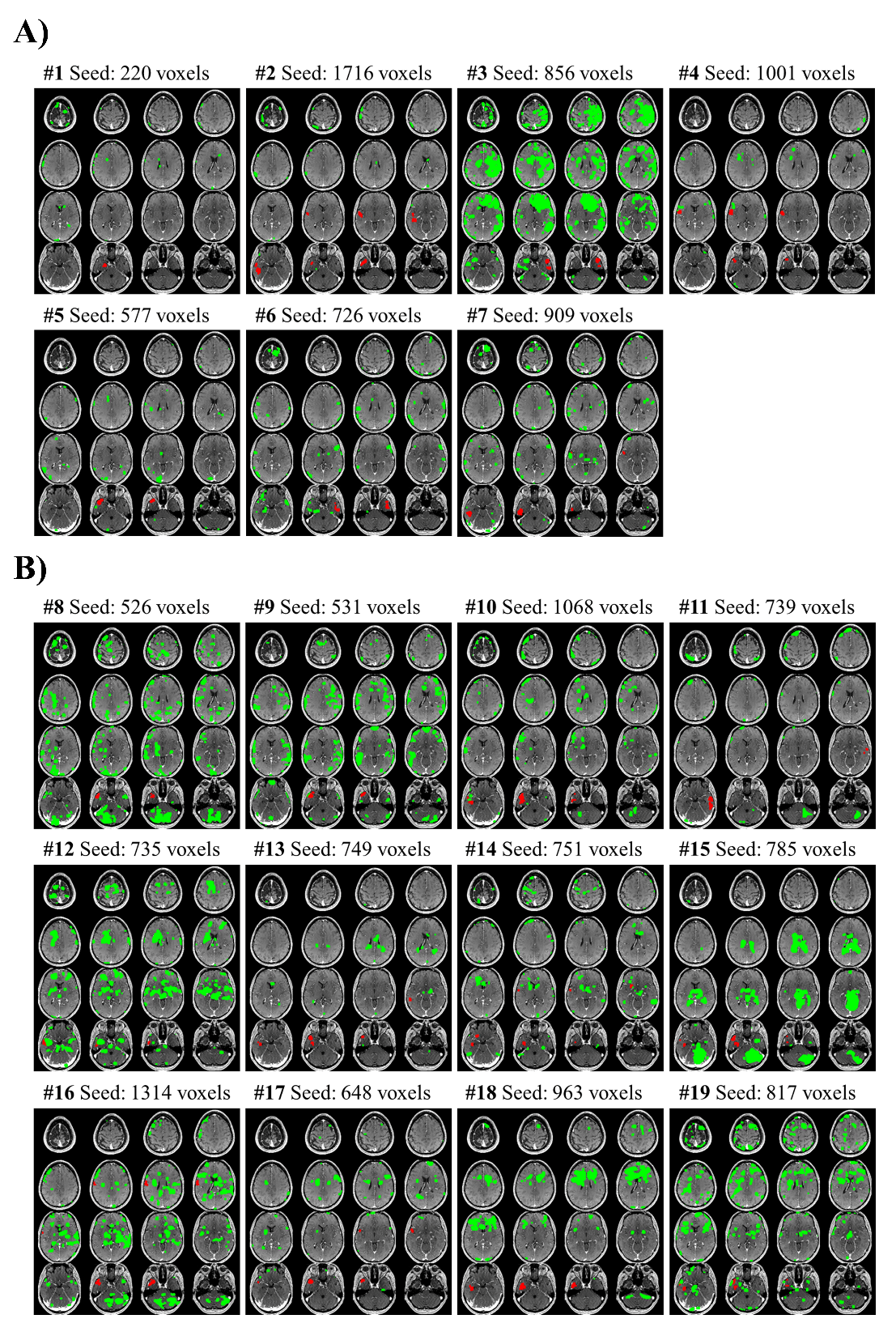
**

*Legend:* All hypothesized epileptogenic seed volumes are shown in red for each patient and the seed volume size is also given. The green volumes represent the negatively correlated epilepsy network for each patient. A) Hypothesized epileptogenic zones and negatively correlated epilepsy networks for all patients who were not seizure free. B) Seed volumes and negatively correlated epilepsy network are shown for all patients that were seizure free.

**Supplementary Figure 2:**


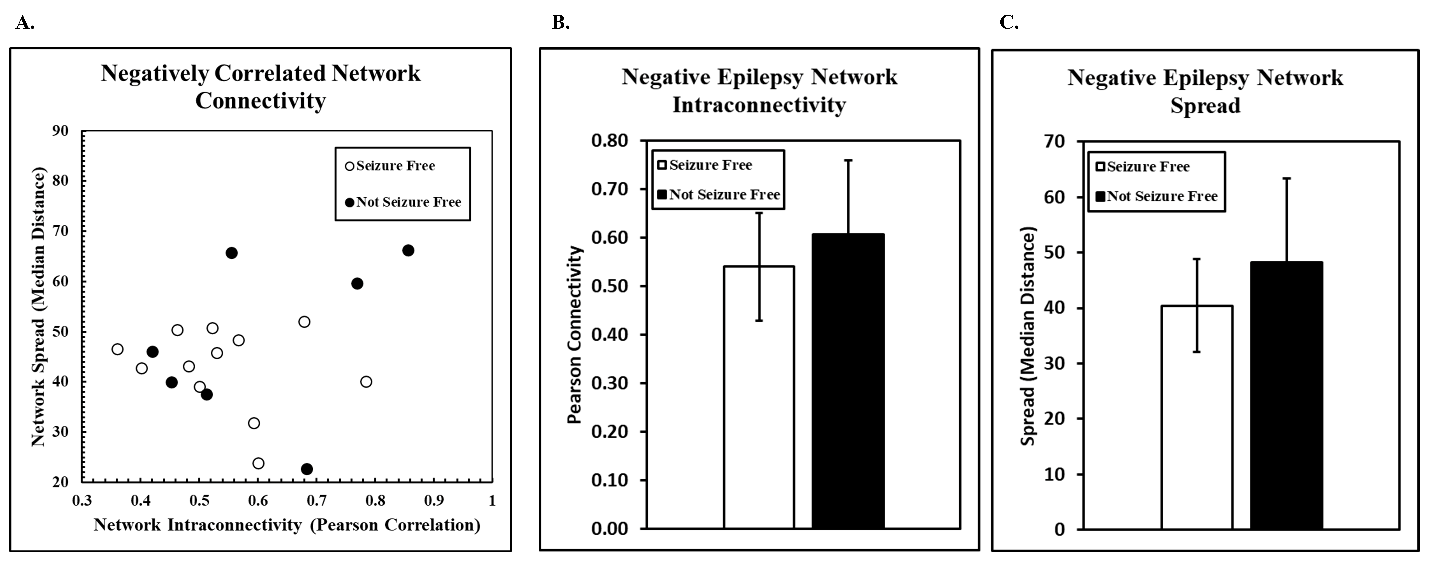


*Legend:* A randomly generated ROI was used to create a negatively correlated network using the same algorithm as described in the methods section. A. No significant relationship between network spread and intraconnectivity is seen when the negatively correlated epilepsy network maps are analyzed. B. No significant difference in network intraconnectivity or C. spread was found between patients that were seizure free after surgery and those who were not.
